# Ferritin, transferrin, and transferrin receptor in relation to metabolic obesity phenotypes: Findings from the China Health and Nutrition Survey

**DOI:** 10.3389/fpubh.2022.922863

**Published:** 2022-08-26

**Authors:** Ziyang Ren, Xingqi Cao, Chenxi Li, Jingyun Zhang, Xueqin Li, Peige Song, Yimin Zhu, Zuyun Liu

**Affiliations:** ^1^The Key Laboratory of Intelligent Preventive Medicine of Zhejiang Province, Center for Clinical Big Data and Analytics of the Second Affiliated Hospital and Department of Big Data in Health Science School of Public Health, Zhejiang University School of Medicine, Hangzhou, China; ^2^School of Public Health and Women's Hospital, Zhejiang University School of Medicine, Hangzhou, China; ^3^Department of Epidemiology and Biostatistics, School of Public Health, Zhejiang University School of Medicine, Hangzhou, China

**Keywords:** iron indicator, age, metabolic heterogeneity, overweight, obesity, sex-stratified

## Abstract

**Background:**

This study aimed to explore the relationship between iron markers and metabolic obesity phenotypes and the role of age.

**Methods:**

Data were from the China Health and Nutrition Survey 2009. Metabolic obesity phenotypes included metabolically healthy with normal weight (MHNW), metabolically unhealthy with normal weight (MUNW), metabolically healthy with overweight/obesity (MHO), and metabolically unhealthy with overweight/obesity (MUO). Iron markers including ferritin, transferrin, and soluble transferrin receptor were calculated as Log and quartered. The linear regression and multinomial logistic regression were used to explore the association of iron markers with age and metabolic obesity phenotypes, respectively.

**Results:**

Ferritin was linearly related with age, with β (95% confidence interval, CI) of 0.029 (0.027 to 0.032) and -0.005 (-0.007 to -0.002) for women and men. Transferrin was negatively associated with age in both men and women (β < -0.011). Furthermore, compared with participants in the quartile 1 ferritin group, those in the quartile 4 had increased odds of MUNW, MHO, and MUO, with odds ratio and 95% confidence interval (OR, 95% CI) of 3.06 (2.20 to 4.25), 1.66 (1.35 to 2.05), and 5.27 (4.17 to 6.66). Transferrin showed similar relationships with MUNW, MUO, and MHO; whereas transferrin receptor showed no significance. We also found joint associations of ferritin and transferrin with MUNW, MUO, and MHO. The interactive effect of ferritin and transferrin on MUO was significant (*P* = 0.015).

**Conclusion:**

Increased ferritin and transferrin were associated with MUNW, MHO, and MUO. Age should be considered when investigating iron.

## Introduction

Iron is a nutritionally essential trace element that is absorbed mostly through the gastrointestinal tract or released by senescent red blood cells (1), then bound by transferrin and transported to the cell surface, where it attaches to transferrin receptors and enters the cell (2). Iron is stored as ferritin, which is found largely in the liver and red blood cells (3). Iron is crucial for a variety of physiological processes, such as oxygen transport and protein repair (4, 5) and can also fluctuate due to factors such as inflammation levels and disease states (6). Three blood-based biomarkers are widely used to measure iron status: ferritin, transferrin, and transferrin receptor (7–9). These iron markers may change due to age-related physiological dysregulations (10). According to a previous study, serum ferritin grew with age in women but had an inverted U-shaped relationship in men (11). Since anemia and iron status are tightly associated (12), the impact of anemia should be taken into account while performing iron biomarker studies. However, sex-stratified associations of age with ferritin, transferrin, and transferrin receptors after excluding the potential effect of anemia remain unknown.

Obesity is a risk factor for a wide range of disorders, and its incidence has risen globally over the last 50 years (13). Given that the traditional obesity markers, such as the body mass index (BMI), cannot describe body fat distribution, metabolic abnormalities were used to further distinguish metabolic heterogeneity of obesity, with metabolically healthy obesity (MHO) being the healthier phenotype vs. metabolically unhealthy obesity (MUO) (14). Individuals with MHO, despite developing elevated BMI, possessed normal serum lipid, blood glucose, insulin sensitivity, and waist circumference (WC), and are less likely to develop cardiovascular illnesses than those with MUO (15–17). Currently, abnormal iron levels, such as iron overload and deficiency, have been found to be related to metabolic abnormalities like metabolic syndrome and type 2 diabetes mellitus (T2DM) (18, 19). A recent study also found that increased transferrin receptor levels were associated with lower risks of type 2 diabetes mellitus in non-obese subjects but increased risks in obese subjects (20), suggesting the importance of considering metabolic obesity phenotypes when studying markers. However, limited studies have assessed the association of iron markers with metabolic obesity phenotypes. Kim et al. (21) found that serum ferritin was positively associated with metabolically unhealthy but normal weight (MUNW) in the Korean population. Suárez-Ortegón et al. (22) also found that higher ferritin was associated with MUO in prepubertal children. However, the sex-stratified associations of ferritin, transferrin, and transferrin receptor with various metabolic obesity phenotypes in the general Chinese population remain unknown. Since the Chinese have a distinct dietary structure (23), it is of interest to explore the association between iron markers and metabolic obesity phenotypes in the Chinese population.

Therefore, we conducted this study to explore the relationship between age and the three iron markers (i.e., ferritin, transferrin, and transferrin receptor), as well as the associations of these iron markers with different metabolic obesity phenotypes in the general Chinese population after excluding the effect of anemia.

## Materials and methods

### Study population

The China Health and Nutrition Survey (CHNS) is an ongoing and prospective household-based cohort study that began in 1989 and investigates a wide range of socio-economic, demographic, nutritional, and health-related information. A multistage random cluster sampling design was applied to select a sample of roughly 7,200 households from 15 provinces across China, with a total population of over 30,000 people. Detailed information on the CHNS was described elsewhere (24, 25). The study was approved by the Institutional Review Boards at the University of North Carolina at Chapel Hill and the National Institute of Nutrition and Food Safety, and all participants completed a written informed consent form.

**Figure 1:**
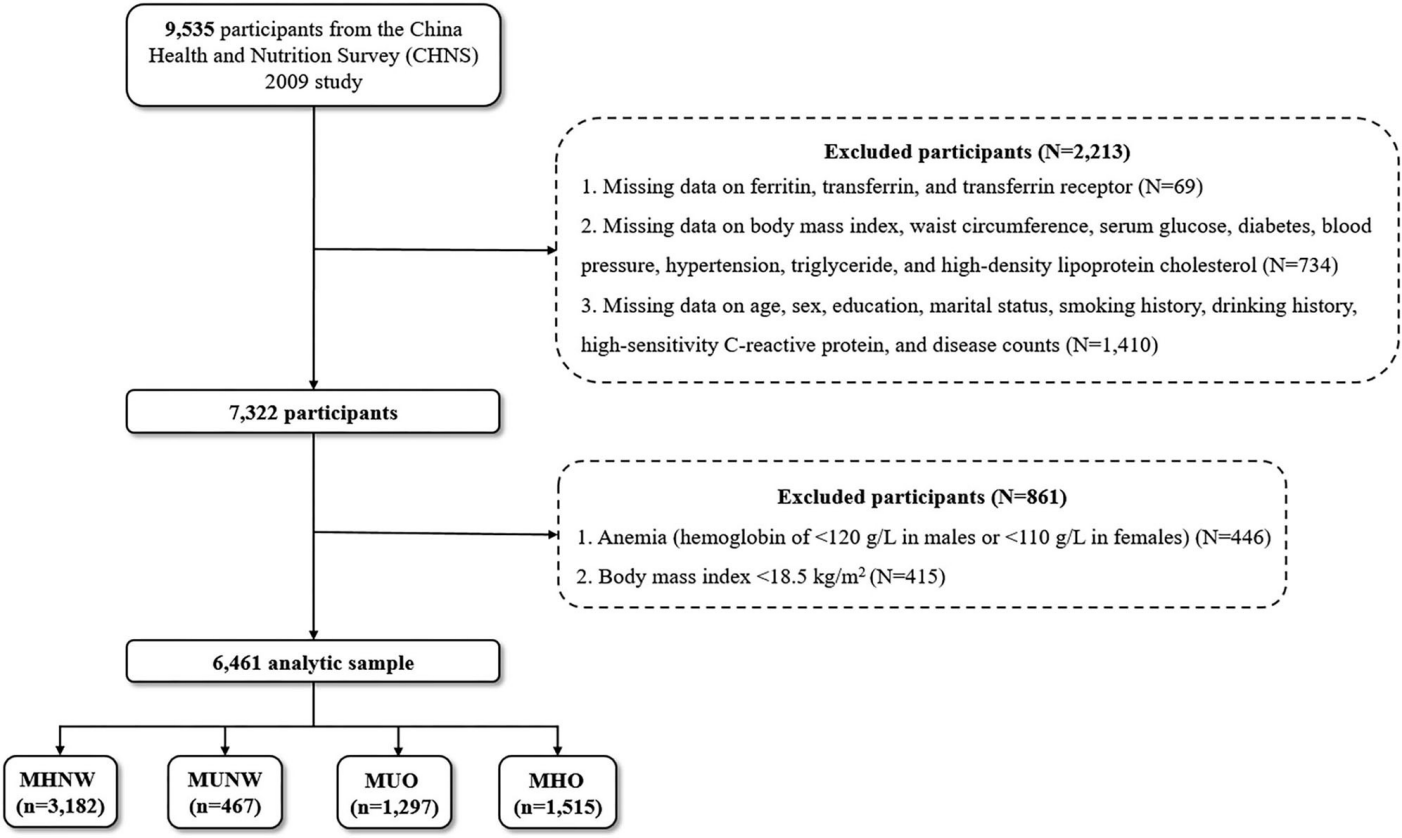
Flow chart of study participants. MHNW, metabolically healthy with normal weight; MUNW, metabolically unhealthy with normal weight; MUO, metabolically unhealthy with overweight/obesity; MHO, metabolically healthy with overweight/obesity.

Data from the CHNS 2009 (with blood-based biomarkers available) were utilized. Participants with missing data on ferritin, transferrin, and transferrin receptor (*N* = 69); with missing data on BMI, WC, serum glucose, diabetes, blood pressure, hypertension, triglyceride (TG), and high-density lipoprotein cholesterol (HDL-C) (*N* = 734); with missing data on age, sex, education, marital status, smoking history, drinking history, high-sensitivity C-reactive protein (hs-CRP), and disease counts (*N* = 1410); and who had anemia or with a BMI of <18.5 kg/m^2^ were excluded, leaving a final analytic sample of 6,461 Chinese participants of all age ranges (Figure 1).

### Measurement of blood-based biomarkers

Biomarker levels were determined using an overnight fasting (12 h) blood sample (12 ml). All samples were centrifuged at 3,000 ×*g* at room temperature for 10 min before being separated into 9 aliquots. Other plasma and serum samples were kept in – 80°C freezers for laboratory analysis, except those for field testing. The samples were subsequently assessed at the Beijing National Central Laboratory (Medical Laboratory Accreditation Certificate ISO 15189:2007) under stringent quality control (26, 27).

Radioimmunology was used to test serum ferritin on a gamma counter XH-6020 made by the North Institute of Bio-Tech in China. Serum transferrin and soluble transferrin receptor were measured using nephelometry on Siemens BNP made by Siemens, Germany. Since serum ferritin, transferrin, and soluble transferrin receptor did not follow a normal distribution, they were calculated as log (ferritin), log (transferrin), and log (transferrin receptor) and allocated by quartiles.

Fasting blood glucose (FBG) measurements (enzymatic method) and regular blood tests were conducted at local hospitals. TG and HDL-C were measured using the enzymatic method and Glycerol phosphate oxiase-peroxidase anti peroxidase (GPO-PAP) on a Hitachi 7600 (Kyowa, Japan). Serum hs-CRP was measured using immunoturbidimetric on a Hitachi 7600 made by Denka Seiken, Japan.

### Assessment and definition of metabolically unhealthy and overweight/obesity

Universally trained interviewers (physicians and nutritionists) conducted anthropometric measurements including weight, height, WC, systolic blood pressure (SBP), and diastolic blood pressure (DBP) in 2009 (28).

Metabolically unhealthy included three or more of the following components (29, 30):

1. Central Obesity: WC of ≥90 cm for men, of ≥85 cm for women.
2. Diabetes: FBG of ≥5.6 Mmol/L and/or Diagnosed as Diabetes or Diabetes Therapy.
3. Hypertension: Blood Pressure of ≥130/85 MmHg and/or Diagnosed as Hypertension or Hypertension Therapy.
4. High TG: TG of ≥1.70 Mmol/L.
5. Low HDL-C: HDL-C of <1.00 Mmol/L for men and <1.30 Mmol/L for Women.

We calculated BMI as weight (kg) divided by the square of height (m^2^). BMI was defined as obesity (≥28 kg/m^2^), overweight (<28 kg/m^2^ and ≥24 kg/m^2^), and normal weight (<24 kg/m^2^ and ≥18.5 kg/m^2^), which is widely used in the Chinese population (31, 32).

Metabolically healthy with normal weight was defined as MHNW; metabolically unhealthy with normal weight was defined as MUNW; metabolically unhealthy with overweight/obesity was defined as MUO; and metabolically healthy with overweight/obesity was defined as MHO.

### Measurement of covariates

Information on age, sex, education, marital status, residence, smoking history, and drinking history was collected through questionnaires in 2009. Education was categorized into four levels (no schooling, primary school, middle school, and high school or more). Marital status was dichotomized as currently married or others. The residence was defined by the community in which participants lived, and it was further divided into urban or rural. Smoking history was classified as nonsmokers or smokers (former or current smokers), respectively. Drinking history was also classified as nondrinkers or drinkers (former or current drinkers). Disease counts were the sum of hypertension, diabetes mellitus, myocardial infarction, stroke, hip fracture, asthma, and cancer, which were further classified as 0, 1, 2, or more.

### Statistical analysis

The medians and interquartile ranges (IQRs) for non-normally distributed continuous variables, and No. (%) for categorical variables were employed to compare baseline characteristics of included participants by different metabolic obesity phenotypes.

First, to examine group comparisons for each variable, Wilcoxon rank sum tests for continuous variables and Chi-squared test for categorical variables were utilized. We also plotted sex-stratified trends of log (ferritin), log (transferrin), and log (transferrin receptor) with age. Since the values of three iron indicators after Log transform were not normally distributed, we further used the Blom transform to normalize them and generate their ranks. Multivariable linear regression was used to analyze the associations of age with the ranks of three iron markers. Model 1 was sex-adjusted. Model 2 further adjusted for smoking history, drinking history, education, residence, marital status, BMI, hs-CRP, and disease counts. In the sex-stratified analysis, the same models (except sex) were used.

Second, we utilized multinomial logistic regression models to examine the relationship between the three iron markers and metabolic obesity phenotypes with MHNW assigned as the reference for MUNW, MUO, and MHO. The test of the proportional odds assumption in PROC LOGISTIC was significant (*P* < 0.05). Meanwhile, the exposures (i.e., ferritin, transferrin, and transferrin receptor) were quartered, and quartile 1 was assigned as the reference for quartiles 2–4. Odds ratios (ORs) and 95% confidence intervals (95% CIs) were reported. Model 1 was sex-adjusted. Model 2 further adjusted for age based on Model 1. Model 3 further adjusted for smoking history, drinking history, education, residence, marital status, hs-CRP, and disease counts based on Model 2. These models were then used to conduct sex-stratified analysis (except sex). We further defined metabolically healthy more rigorously to assure the robustness of our findings in the sensitivity analysis. Metabolically healthy was defined as possessing none of the above five metabolic abnormalities, metabolically sub-healthy was defined as one or two abnormalities, and metabolically unhealthy was defined as three or more abnormalities. Hence, we redefined metabolically healthy with normal weight as MHNW, metabolically sub-healthy with normal weight as MSHNW; metabolically unhealthy with normal weight as MUNW, metabolically healthy with overweight/obesity as MHO, metabolically sub-healthy with overweight/obesity as MSHO, and metabolically unhealthy with overweight/obesity as MUO, and explored their associations with iron markers.

Third, since ferritin and transferrin were significantly associated with metabolic obesity phenotypes, we divided the study population into high and low according to 50% of the population based on ferritin or transferrin respectively and combined them into four iron groups: low ferritin and low transferrin, low ferritin and high transferrin, high ferritin and low transferrin, and high ferritin and high transferrin. Multinomial logistic regression was used to determine the relationship between the four joint iron groups and metabolic obesity phenotypes. We used the same three models above (Model 1, sex-adjusted; Model 2, age- and sex-adjusted; Model 3, fully adjusted). Sex-stratified analysis was also conducted. We also explored the interactive effect of ferritin and transferrin on metabolic obesity phenotypes using multinomial logistic regression with Model 3 adjusted.

A two-sided *P* < 0.05 was considered statistically significant. All analyses were conducted using SAS statistical software (version 9.4; SAS Institute Inc., Cary, NC, USA).

## Results

The baseline characteristics of included participants were summarized in Table 1. Of the 6,461 individuals included, 3,182 (49.2%) were defined as MHNW, 467 (7.2%) as MUNW, 1,297 (20.1%) as MUO, and 1,515 (23.4%) were classified as MHO. Age, sex, education, marital status, residence, smoking history, drinking history, disease counts, BMI, WC, SBP, DBP, FBG, TG, HDL-C, hs-CRP, log (ferritin), and log (transferrin) all had statistically significant differences across the four groups (all *P* values < 0.05).

**Table 1:** Basic characteristics of study participants in the CHNS 2009 (*N* = 6,461).

	**MHNW**	**MUNW**	**MHO**	**MUO**	***P* value**
Participants	3,182 (49.3)	467 (7.2)	1,515 (23.5)	1,297 (20.1)
Age, year	48.00 (37.00–59.00)	60.00 (50.00–68.00)*	56.00 (45.00–63.00)*	49.00 (39.00–59.00)	<0.001
Sex					<0.001
Female	1,661 (52.2)	294 (63.0)*	806 (53.2)	717 (55.3)
Male	1,521 (47.8)	173 (37.0)	709 (46.8)	580 (44.7)
Education					<0.001
No schooling	781 (24.5)	168 (36.0)*	350 (23.1)	380 (29.3)*
Primary school	697 (21.9)	110 (23.6)	334 (22.1)	303 (23.4)
Middle school	1,248 (39.2)	130 (27.8)	603 (39.8)	463 (35.7)
High school or more	456 (14.3)	59 (12.6)	228 (15.1)	151 (11.6)
Marital status					<0.001
Currently married	2,674 (84.0)	386 (82.7)	1,352 (89.2)*	1,119 (86.3)
Others	508 (16.0)	81 (17.3)	163 (10.8)	178 (13.7)
Residence					<0.001
Urban	806 (25.3)	177 (37.9)*	420 (27.7)	439 (33.9)*
Rural	2376 (74.7)	290 (62.1)	1095 (72.3)	858 (66.2)
Smoking history					<0.001
Nonsmoker	2,208 (69.4)	363 (77.7)*	1,155 (76.2)*	964 (74.3)*
Smoker	974 (30.6)	104 (22.3)	360 (23.8)	333 (25.7)
Drinking history					0.001
Nondrinker	2,118 (66.6)	362 (77.5)*	1,005 (66.3)	878 (67.7)
Drinker	1,064 (33.4)	105 (22.5)	510 (33.7)	419 (32.3)
Disease counts					<0.001
0	2,780 (87.4)	306 (65.5)*	1,253 (82.7)*	788 (60.8)*
1	348 (10.9)	110 (23.6)	222 (14.7)	376 (29.0)
2 or more	54 (1.7)	51 (10.9)	40 (2.6)	133 (10.3)
Body mass index, kg/m^2^	21.47 (20.30–22.62)	22.56 (21.41–23.37)*	25.65 (24.69–27.02)*	27.12 (25.49–29.00)*	<0.001
Waist circumference, cm	77.30 (72.80–82.00)	85.00 (80.00–90.00)*	87.5 (83.00–93.00)*	94.00 (89.00–99.00)*	<0.001
Systolic blood pressure, mmHg	119.33 (110.00–128.67)	132.00 (120.67–146.67)*	120.67 (114.00–131.00)*	133.33 (122.00–148.67)*	<0.001
Diastolic blood pressure, mmHg	78.67 (70.00–82.00)	85.00 (79.33–90.33)*	80.00 (75.33–86.00)*	87.33 (80.00–92.67)*	<0.001
Fasting blood glucose, mmol/L	4.94 (4.60–5.34)	5.83 (5.40–6.72)*	5.04 (4.69–5.39)*	5.73 (5.16–6.55)*	<0.001
Triglycerides, mmol/L	1.04 (0.75–1.46)	2.25 (1.76–3.13)*	1.22 (0.88–1.62)*	2.33 (1.74–3.29)*	<0.001
High density lipoprotein cholesterol, mmol/L	1.49 (1.28–1.74)	1.16 (0.99–1.35)*	1.39 (1.19–1.49)*	1.15 (0.98–1.36)*	<0.001
High-sensitivity C–reactive protein, mg/L	1.00 (0–2.00)	2.00 (1.00–3.00)*	1.00 (0–1.00)*	1.00 (1.00–2.00)*	<0.001
Log (ferritin), ng/mL	4.28 (3.59–4.86)	4.60 (4.04–5.23)*	4.36 (3.71–4.99)*	4.71 (4.14–5.36)*	<0.001
Log (transferrin), mg/dL	5.61 (5.51–5.74)	5.66 (5.53–5.78)*	5.66 (5.55–5.77)*	5.68 (5.57–5.79)*	<0.001
Log (transferrin receptor), mg/L	0.28 (0.08–0.48)	0.31 (0.07–0.49)	0.29 (0.08–0.48)	0.30 (0.08–0.51)	0.496

CHNS, China Health and Nutrition Survey; MHNW, metabolically healthy with normal weight; MUNW, metabolically unhealthy with normal weight; MHO, metabolically healthy with overweight/obesity; MUO, metabolically unhealthy with overweight/obesity. Values are presented as median (interquartile range) or No. (%).

P-value was calculated by Chi-square test or Wilcoxon rank sum test.

^*^Means P < 0.05 from MUNW, MUO, and MHO compared to MHNW, respectively.

Supplementary Figure 1 shows an S-shaped change in ferritin with age in women (decreasing until approximately 32 years of age and increasing rapidly and then slowly thereafter) but a linear decrease with age in men. Transferrin, on the contrary, decreases linearly with age in both men and women. However, we did not find a significant relationship between the transferrin receptor and age. According to Supplementary Table 1, age was significantly related to the rank of log (ferritin) (β = 0.013, 95% CI: 0.011 to 0.015 in all participants; β = 0.029, 95% CI: 0.027 to 0.032 in women; and β = − 0.005, 95% CI: −0.007 to −0.002 in men; all *P* values < 0.001). Meanwhile, age was negatively associated with the rank of log (transferrin) (β = − 0.013, 95% CI: −0.014 to −0.011 in all participants; β = −0.014, 95% CI: −0.017 to −0.011 in women; and β = -0.011, 95% CI: −0.014 to −0.008 in men; all *P* values <0.001). The relationship between age and the rank of log (transferrin receptor) was non-significant.

In the multinomial logistic analysis, when compared to those who were defined as MHNW, we found significant relationships of higher log (ferritin) with MUNW (OR = 2.95, 95% CI: 2.14 to 4.06 for quartile 4; *P* for trend <0.001), MHO (OR = 1.56, 95% CI: 1.27 to 1.91 for quartile 4; *P* for trend <0.001), and MUO (OR = 4.93, 95% CI: 3.93 to 6.17 for quartile 4; *P* for trend <0.001) after adjusting for sex (not in the sex-stratified analysis) and age. In women, nevertheless, we found no significant relationships between higher log (ferritin) and MHO (OR = 1.11, 95% CI: 0.84 to 1.47 for the quartile 4). In Model 3, we adjusted for additional factors and found that the relationships remained similar. After Model 3 adjustment in the total population, significant relationships of log(transferrin) with MUNW (OR = 2.54, 95% CI: 1.91 to 3.39 for the quartile 4; *P* for trend <0.001), MHO (OR = 1.82, 95% CI: 1.52 to 2.18 for the quartile 4; *P* for trend <0.001), and MUO (OR = 3.31, 95% CI: 2.70 to 4.07 for the quartile 4; *P* for trend <0.001) were found, which was also found in sex-stratified analyses (all *P* for trend <0.001). However, in all models, there was no evidence of a significant association between log (transferrin receptor) and metabolic obesity phenotypes (Tables 2, 3, and Supplementary Table 2). The results of the sensitivity analysis were similar to our main findings, but the relationship between ferritin and MHO became non-significant when compared with MHNW (Supplementary Table 3).

**Table 2:** Odds ratios and 95% CIs of different metabolic obesity phenotypes by ferritin levels in CHNS 2009.

	**MUNW**			**MHO**			**MUO**		
	**Model 1**	**Model 2**	**Model 3**	**Model 1**	**Model 2**	**Model 3**	**Model 1**	**Model 2**	**Model 3**
**Overall (N** **=** **6,461)**									
Quartile 1	Reference	Reference	Reference	Reference	Reference	Reference	Reference	Reference	Reference
Quartile 2	**1.92 (1.41 to 2.61)**	1.26 (0.92 to 1.73)	1.28 (0.93 to 1.76)	1.18 (0.99 to 1.40)	1.16 (0.97 to 1.39)	**1.21 (1.01 to 1.45)**	**2.20 (1.78 to 2.71)**	**1.78 (1.43 to 2.20)**	**1.83 (1.47 to 2.29)**
Quartile 3	**2.90 (2.13 to 3.93)**	**1.71 (1.25 to 2.34)**	**1.73 (1.26 to 2.38)**	1.17 (0.97 to 1.40)	1.15 (0.95 to 1.39)	**1.22 (1.00 to 1.48)**	**2.95 (2.38 to 3.65)**	**2.26 (1.82 to 2.82)**	**2.34 (1.86 to 2.94)**
Quartile 4	**4.94 (3.60 to 6.79)**	**2.95 (2.14 to 4.06)**	**3.06 (2.20 to 4.25)**	**1.57 (1.29 to 1.92)**	**1.56 (1.27 to 1.91)**	**1.66 (1.35 to 2.05)**	**6.33 (5.08 to 7.89)**	**4.93 (3.93 to 6.17)**	**5.27 (4.17 to 6.66)**
P for trend	<0.001	<0.001	<0.001	<0.001	<0.001	<0.001	<0.001	<0.001	<0.001
**Female (N** **=** **3,478)**									
Quartile 1	Reference	Reference	Reference	Reference	Reference	Reference	Reference	Reference	Reference
Quartile 2	1.33 (0.87 to 2.03)	0.86 (0.56 to 1.35)	0.90 (0.57 to 1.40)	1.08 (0.86 to 1.35)	1.02 (0.81 to 1.28)	1.06 (0.84 to 1.34)	**1.54 (1.15 to 2.05)**	1.21 (0.90 to 1.63)	1.26 (0.93 to 1.72)
Quartile 3	**2.96 (2.00 to 4.36)**	1.35 (0.89 to 2.05)	1.32 (0.86 to 2.02)	**1.29 (1.02 to 1.63)**	1.14 (0.89 to 1.46)	1.15 (0.89 to 1.48)	**2.87 (2.18 to 3.79)**	**1.80 (1.34 to 2.42)**	**1.74 (1.28 to 2.37)**
Quartile 4	**4.45 (3.03 to 6.52)**	**1.56 (1.02 to 2.39)**	1.49 (0.96 to 2.30)	**1.33 (1.04 to 1.70)**	1.11 (0.84 to 1.47)	1.10 (0.83 to 1.46)	**5.03 (3.83 to 6.59)**	**2.66 (1.96 to 3.60)**	**2.48 (1.81 to 3.41)**
P for trend	<0.001	0.008	0.021	0.009	0.365	0.418	<0.001	<0.001	<0.001
**Male (N** **=** **2,983)**									
Quartile 1	Reference	Reference	Reference	Reference	Reference	Reference	Reference	Reference	Reference
Quartile 2	1.26 (0.76 to 2.07)	1.37 (0.83 to 2.28)	1.42 (0.85 to 2.36)	1.13 (0.88 to 1.45)	1.12 (0.88 to 1.43)	1.12 (0.87 to 1.44)	**1.65 (1.21 to 2.25)**	**1.71 (1.25 to 2.34)**	**1.82 (1.32 to 2.52)**
Quartile 3	**1.66 (1.03 to 2.69)**	**1.81 (1.12 to 2.95)**	**2.00 (1.22 to 3.27)**	1.18 (0.92 to 1.52)	1.17 (0.91 to 1.51)	1.21 (0.94 to 1.56)	**2.14 (1.58 to 2.90)**	**2.23 (1.64 to 3.02)**	**2.48 (1.81 to 3.41)**
Quartile 4	**3.48 (2.21 to 5.48)**	**4.07 (2.57 to 6.46)**	**4.23 (2.64 to 6.78)**	**1.69 (1.30 to 2.18)**	**1.66 (1.29 to 2.15)**	**1.69 (1.30 to 2.20)**	**4.87 (3.63 to 6.54)**	**5.19 (3.86 to 6.98)**	**5.65 (4.14 to 7.71)**
*P* for trend	<0.001	<0.001	<0.001	<0.001	<0.001	<0.001	<0.001	<0.001	<0.001

CI, confidence interval; MHNW, metabolically healthy with normal weight; MUNW, metabolically unhealthy with normal weight; MHO, metabolically healthy with overweight/obesity; MUO, metabolically unhealthy with overweight/obesity.

The reference of multinomial logistic models was MHNW. Model 1 adjusted for sex. Model 2 further adjusted for age based on Model 1. Model 3 further adjusted for smoking history, drinking history, education, residence, marital status, high-sensitivity C-reactive protein, and disease counts based on Model 2.

The bold values indicate the statistically significant.

**Table 3:** Odds ratios and 95% CIs of different metabolic obesity phenotypes by transferrin levels in CHNS 2009.

	**MUNW**			**MHO**			**MUO**		
	**Model 1**	**Model 2**	**Model 3**	**Model 1**	**Model 2**	**Model 3**	**Model 1**	**Model 2**	**Model 3**
**Overall (N** **=** **6,461)**									
Quartile 1	Reference	Reference	Reference	Reference	Reference	Reference	Reference	Reference	Reference
Quartile 2	1.07 (0.81 to 1.42)	1.29 (0.97 to 1.71)	**1.37 (1.03 to 1.83)**	**1.21 (1.01 to 1.44)**	**1.23 (1.03 to 1.47)**	**1.25 (1.05 to 1.49)**	**1.51 (1.24 to 1.83)**	**1.69 (1.39 to 2.06)**	**1.78 (1.45 to 2.18)**
Quartile 3	1.25 (0.95 to 1.65)	**1.73 (1.30 to 2.30)**	**1.86 (1.39 to 2.49)**	**1.49 (1.26 to 1.78)**	**1.55 (1.30 to 1.84)**	**1.58 (1.32 to 1.88)**	**1.94 (1.60 to 2.35)**	**2.39 (1.96 to 2.91)**	**2.55 (2.08 to 3.12)**
Quartile 4	**1.52 (1.16 to 1.99)**	**2.37 (1.78 to 3.15)**	**2.54 (1.91 to 3.39)**	**1.72 (1.45 to 2.05)**	**1.81 (1.51 to 2.16)**	**1.82 (1.52 to 2.18)**	**2.38 (1.97 to 2.88)**	**3.16 (2.59 to 3.84)**	**3.31 (2.70 to 4.07)**
*P* for trend	0.001	<0.001	<0.001	0.001	<0.001	<0.001	0.001	<0.001	<0.001
**Female (N** **=** **3,478)**									
Quartile 1	Reference	Reference	Reference	Reference	Reference	Reference	Reference	Reference	Reference
Quartile 2	1.13 (0.79 to 1.61)	**1.53 (1.06 to 2.23)**	**1.52 (1.04 to 2.22)**	1.17 (0.92 to 1.49)	1.24 (0.97 to 1.58)	1.23 (0.96 to 1.58)	**1.29 (1.00 to 1.67)**	**1.62 (1.24 to 2.12)**	**1.63 (1.23 to 2.16)**
Quartile 3	1.31 (0.92 to 1.86)	**2.12 (1.47 to 3.06)**	**2.18 (1.50 to 3.16)**	**1.28 (1.01 to 1.63)**	**1.41 (1.11 to 1.80)**	**1.41 (1.10 to 1.81)**	**1.52 (1.18 to 1.95)**	**2.17 (1.66 to 2.83)**	**2.27 (1.71 to 3.00)**
Quartile 4	1.36 (0.96 to 1.94)	**2.71 (1.86 to 3.96)**	**2.75 (1.87 to 4.03)**	**1.60 (1.27 to 2.03)**	**1.84 (1.44 to 2.35)**	**1.80 (1.41 to 2.31)**	**1.64 (1.28 to 2.11)**	**2.75 (2.10 to 3.61)**	**2.84 (2.13 to 3.77)**
*P* for trend	0.056	<0.001	<0.001	<0.001	<0.001	<0.001	<0.001	<0.001	<0.001
**Male (N** **=** **2,983)**									
Quartile 1	Reference	Reference	Reference	Reference	Reference	Reference	Reference	Reference	Reference
Quartile 2	1.01 (0.65 to 1.58)	1.15 (0.73 to 1.80)	1.27 (0.80 to 2.02)	1.18 (0.92 to 1.53)	1.17 (0.91 to 1.52)	1.18 (0.91 to 1.53)	**1.74 (1.28 to 2.37)**	**1.85 (1.35 to 2.52)**	**1.91 (1.38 to 2.64)**
Quartile 3	1.10 (0.70 to 1.73)	1.34 (0.85 to 2.13)	1.55 (0.97 to 2.49)	**1.51 (1.18 to 1.95)**	**1.49 (1.16 to 1.93)**	**1.55 (1.19 to 2.02)**	**2.35 (1.73 to 3.18)**	**2.59 (1.90 to 3.52)**	**2.87 (2.08 to 3.96)**
Quartile 4	**1.61 (1.04 to 2.48)**	**1.99 (1.28 to 3.09)**	**2.31 (1.46 to 3.63)**	**1.79 (1.38 to 2.31)**	**1.76 (1.36 to 2.28)**	**1.86 (1.42 to 2.42)**	**3.85 (2.87 to 5.17)**	**4.27 (3.16 to 5.75)**	**4.70 (3.43 to 6.44)**
*P* for trend	0.033	0.002	<0.001	<0.001	<0.001	<0.001	<0.001	<0.001	<0.001

CI, confidence interval; MHNW, metabolically healthy with normal weight; MUNW, metabolically unhealthy with normal weight; MHO, metabolically healthy with overweight/obesity; MUO, metabolically unhealthy with overweight/obesity.

*P*-value for trend across categories, based on category midpoint. The reference of multinomial logistic models was MHNW. Model 1 adjusted for sex. Model 2 further adjusted for age based on Model 1. Model 3 further adjusted for smoking history, drinking history, education, residence, marital status, high-sensitivity C-reactive protein, and disease counts based on Model 2.

The bold values indicate the statistically significant.

We also found significant associations of low ferritin and high transferrin (OR = 1.71, 95% CI: 1.25 to 2.35), high ferritin and low transferrin (OR = 1.80, 95% CI: 1.32 to 2.46), and high ferritin and high transferrin (OR = 4.26, 95% CI: 3.08 to 5.89) with MUNW when compared to low ferritin and low transferrin. Moreover, the relationships of MUO with low ferritin and high transferrin (OR = 1.83, 95% CI: 1.47 to 2.28), high ferritin and low transferrin (OR = 2.06, 95% CI: 1.65 to 2.56), and high ferritin and high transferrin (OR = 5.95, 95% CI: 4.73 to 7.48) were significant. Similar results were also found among the relationships of low ferritin and high transferrin (OR = 1.42, 95% CI: 1.19 to 1.69) and high ferritin and high transferrin (OR = 2.09, 95% CI: 1.71 to 2.56) with MHO when compared to low ferritin and low transferrin (Table 4). The interactive effect of ferritin and transferrin on MUO in the total population was also found (*P* for interaction = 0.015), as shown in Table 4.

**Table 4:** Odds ratios and 95% CIs of different metabolic obesity phenotypes by four iron groups in CHNS 2009.

	**MUNW**	**MHO**	**MUO**
	**OR (95% CI)**		
**Overall**			
Low ferritin and low transferrin	Reference	Reference	Reference
Low ferritin and high transferrin	**1.71 (1.25 to 2.35)**	**1.42 (1.19 to 1.69)**	**1.83 (1.47 to 2.28)**
High ferritin and low transferrin	**1.80 (1.32 to 2.46)**	1.20 (0.99 to 1.44)	**2.06 (1.65 to 2.56)**
High ferritin and high transferrin	**4.26 (3.08 to 5.89)**	**2.09 (1.71 to 2.56)**	**5.95 (4.73 to 7.48)**
*P* for interaction	0.759	0.953	0.015
**Females**			
Low ferritin and low transferrin	Reference	Reference	Reference
Low ferritin and high transferrin	**1.77 (1.23 to 2.53)**	**1.47 (1.19 to 1.81)**	**1.89 (1.46 to 2.45)**
High ferritin and low transferrin	1.22 (0.82 to 1.82)	1.06 (0.80 to 1.39)	**1.51 (1.12 to 2.04)**
High ferritin and high transferrin	**3.12 (2.06 to 4.73)**	**1.51 (1.09 to 2.09)**	**3.85 (2.79 to 5.31)**
*P* for interaction	0.550	0.200	0.576
**Males**			
Low ferritin and low transferrin	Reference	Reference	Reference
Low ferritin and high transferrin	1.91 (0.94 to 3.90)	**1.41 (1.01 to 1.96)**	**2.13 (1.34 to 3.38)**
High ferritin and low transferrin	**2.69 (1.51 to 4.82)**	1.18 (0.89 to 1.55)	**2.73 (1.85 to 4.01)**
High ferritin and high transferrin	**5.11 (2.78 to 9.41)**	**2.14 (1.60 to 2.86)**	**7.90 (5.32 to 11.73)**
*P* for interaction	0.282	0.568	0.136

CI, confidence interval; MHNW, metabolically healthy with normal weight; MUNW, metabolically unhealthy with normal weight; MHO, metabolically healthy with overweight/obesity; MUO, metabolically unhealthy with overweight/obesity.

The reference of multinomial logistic models was MHNW. Odds ratios were adjusted for age, sex, smoking history, drinking history, education, residence, marital status, high-sensitivity C-reactive protein, and disease counts (sex was not included in sex stratified analysis).

The bold values indicate the statistically significant.

## Discussion

In this population-based cross-sectional study, we found a significant association of ferritin and transferrin with MUNW, MHO, and MUO after adjusting for a set of covariates including age, sex, smoking history, drinking history, education, residence, and marital status in both women and men (except sex in sex-stratified analysis). Transferrin receptors, on the contrary, did not exhibit this association. We also found that higher ferritin and transferrin levels were associated with MUNW, MHO, and MUO when compared to low ferritin and low transferrin. The findings suggest an important relationship between iron markers and metabolic obesity.

Our study demonstrated a significant linear relationship between age and serum ferritin, which is consistent with earlier research (11, 33). We also found significant linear relationships between age and serum transferrin in both men and women. People's hematological capacity and liver function deteriorated with age, which might lead to more ferritin release and transferrin inhibition (34). Cellular dystrophy, iron homeostasis dysregulation, and other factors due to aging may also play an important role in elevated ferritin and decreased transferrin (35, 36).

In this study, higher ferritin or transferrin levels were found to be significantly related to MUNW, MHO, and MUO, which is consistent with previous studies (37, 38). Our findings suggest that iron-related biomarkers like ferritin and transferrin are significantly associated with separate obesity or separate metabolic abnormalities. Moreover, it seems that the strength of the associations of ferritin and transferrin with the coexistence of both metabolic abnormalities and obesity was stronger than that for obesity alone (i.e., the ORs of MHO were lower than those of MUO). Iron can block insulin's inhibitory impact on liver glucose synthesis and diminish insulin extraction in the liver, leading to peripheral hyperinsulinemia (39, 40). Insulin, in turn, can stimulate the redistribution of transferrin receptors to the cell surface, resulting in an increase in intracellular iron through ferritin deposition (18, 41). Thus, a vicious cycle between ferritin and insulin develops. Hyperinsulinemia also increases the risk of insulin resistance, as well as lipid and carbohydrate metabolism disorders (42), all of which are metabolic abnormalities and may lead to abnormal iron metabolism.

In particular, we found no significant association between transferrin receptors and metabolic obesity phenotypes. According to prior research, obesity and metabolic abnormalities are significantly associated with chronic inflammatory states (43). However, transferrin receptors are less affected by inflammation, which may partially explain our non-significant findings. In addition, a recent study found that children on a vegetarian diet had significantly higher serum transferrin receptor concentrations than omnivorous children (44), suggesting that dietary iron intake may influence serum iron marker levels. Given the limitations of our data, we cannot clarify the influence of dietary iron intake on our findings. Therefore, more research is needed to validate our findings.

Major findings in this study are in line with a prior study conducted by Han et al. (11). However, we further excluded participants with anemia and focused more on the health consequences of obesity, i.e., distinguishing the metabolic phenotypes of obesity that were not taken into account in Han's study. We also further estimated the relationship of age with transferrin and transferrin receptor, as well as the correlation of iron markers with metabolic obesity phenotypes. Findings from our study demonstrated that it was appropriate to consider age as a confounder when examining the association between iron markers and health outcomes.

To the best of our knowledge, this is the first comprehensive investigation to explore the associations of ferritin, transferrin, and transferrin receptor with age and various metabolic obesity phenotypes in the general population. We found significant linear relationships of age with ferritin and transferrin, which emphasize the importance of adjusting for age when studying iron markers and may contribute to better clinical criteria for iron overload and iron deficiency. Considering that obesity and metabolic abnormalities often coexist, our findings demonstrate that the strength of association of ferritin and transferrin with different metabolic obesity phenotypes varies, which emphasizes the importance of taking full account of the metabolic status of obese subjects when treating their iron dysfunction. Moreover, since the definition of metabolically healthy is controversial (45), we defined metabolically healthy more rigorously (none of the metabolic abnormalities) to assure the robustness of our findings in the sensitivity analysis and found the association between ferritin and MHO became non-significant when compared to MHNW, which may help promote a better understanding of metabolically healthy.

On the contrary, our study remains limited by several shortcomings. First, since this study is cross-sectional in design, the causal relationships between iron markers and metabolic obesity phenotypes remain unclear. Moreover, though the CHNS has stringent technical procedures, our findings may be influenced by the birth cohort effect since our data were from the 2009 wave. Though we have adjusted for several covariates, there were still some potential confounders not measured in this study, such as dietary patterns, medications, and other inflammatory factors like interleukin-6. Finally, other iron indicators like transferrin saturation were not included, which should be explored in future studies.

## Conclusion

We found that there were significant associations of ferritin and transferrin with age and various metabolic obesity phenotypes. Our findings suggest the need to control for age when studying iron and provide population evidence for the clinical treatment of subjects with iron dysfunction of different ages. Moreover, this study emphasizes the importance of focusing on iron dysfunction and unhealthy metabolic status in obese subjects.

## Data availability statement

The original contributions presented in the study are included in the article/Supplementary material, further inquiries can be directed to the corresponding author/s.

## Ethics statement

The studies involving human participants were reviewed and approved by the Institutional Review Board at the University of North Carolina at Chapel Hill, the China-Japan Friendship Hospital, Ministry of Health, and the National Institute for Nutrition and Health, Chinese Center for Disease Control and Prevention granted approval for the China Health and Nutrition Survey. The patients/participants provided their written informed consent to participate in this study.

## Author contributions

ZL designed the study. ZR, XC, and CL managed and analyzed the data. ZR prepared the first draft. XC, CL, JZ, and XL reviewed and edited the manuscript, with comments from PS, YZ, and ZL. All authors were involved in revising the paper, had full access to the data, and gave final approval of the submitted versions.

## Funding

This study was supported by a grant from the National Natural Science Foundation of China (82171584), the 2020 Milstein Medical Asian American Partnership Foundation Irma and Paul Milstein Program for Senior Health project award (ZL), the Fundamental Research Funds for the Central Universities (ZL), the Key Laboratory of Intelligent Preventive Medicine of Zhejiang Province (2020E10004), Leading Innovative and Entrepreneur Team Introduction Program of Zhejiang (2019R01007), Key Research and Development Program of Zhejiang Province (2020C03002), and Zhejiang University Global Partnership Fund (188170–11103). The funders had no role in the study design; data collection, analysis, or interpretation; in the writing of the report; or in the decision to submit the article for publication.

## Conflict of interest

The authors declare that the research was conducted in the absence of any commercial or financial relationships that could be construed as a potential conflict of interest.

## Publisher's note

All claims expressed in this article are solely those of the authors and do not necessarily represent those of their affiliated organizations, or those of the publisher, the editors and the reviewers. Any product that may be evaluated in this article, or claim that may be made by its manufacturer, is not guaranteed or endorsed by the publisher.
