# Supplementary material for: Ferritin, transferrin, and transferrin receptor in relation to metabolic obesity phenotypes: Findings from the China Health and Nutrition Survey
Source: Front Public Health. 2022 Aug 26;10:922863. doi: 10.3389/fpubh.2022.922863 (PMC9459082; doi:10.3389/fpubh.2022.922863)
Supplement: Supplementary file 1 [file Data_Sheet_1.docx]

Supplementary Table 1. The relationships between age and iron indicators.

| **Iron indicators** | | **Model 1** | |  | **Model 2** | |
| --- | --- | --- | --- | --- | --- | --- |
|  |  | **β (95% CI)** | ***P*** |  | **β (95% CI)** | ***P*** |
| Rank of log (ferritin) | | | | | | |
|  | Total | **0.015 (0.014 to 0.017)** | <0.001 |  | **0.013 (0.011 to 0.015)** | <0.001 |
|  | Female | **0.032 (0.030 to 0.034)** | <0.001 |  | **0.029 (0.027 to 0.032)** | <0.001 |
|  | Male | **-0.004 (-0.007 to -0.002)** | <0.001 |  | **-0.005 (-0.007 to -0.002)** | <0.001 |
| Rank of log (transferrin) | | | | | | |
|  | Total | **-0.012 (-0.014 to -0.010)** | <0.001 |  | **-0.013 (-0.014 to -0.011)** | <0.001 |
|  | Female | **-0.015 (-0.017 to -0.012)** | <0.001 |  | **-0.014 (-0.017 to -0.011)** | <0.001 |
|  | Male | **-0.009 (-0.012 to -0.007)** | <0.001 |  | **-0.011 (-0.014 to -0.008)** | <0.001 |
| Rank of log (transferrin receptor) | | | | | | |
|  | Total | 0.001 (-0.001 to 0.003) | 0.243 |  | 0 (-0.002 to 0.002) | 0.800 |
|  | Female | -0.001 (-0.003 to 0.002) | 0.602 |  | -0.001 (-0.004 to 0.001) | 0.336 |
|  | Male | **0.003 (0 to 0.005)** | 0.022 |  | 0.001 (-0.002 to 0.004) | 0.506 |

Notes: Model 1 adjusted for sex (except for sex-stratified analyses). Model 2 further adjusted for smoking history, drinking history, education, residence, marital status, high-sensitivity C-reactive protein, and disease counts based on Model 1.

Supplementary Table 2. Odds ratios and 95% CIs of different metabolic obesity phenotypes by transferrin receptor levels in CHNS 2009.

|  | **MUNW** | | |  | **MHO** | | |  | **MUO** | | |
| --- | --- | --- | --- | --- | --- | --- | --- | --- | --- | --- | --- |
|  | **Model 1** | **Model 2** | **Model 3** |  | **Model 1** | **Model 2** | **Model 3** |  | **Model 1** | **Model 2** | **Model 3** |
| ***Overall (N=6461)*** | | | | | | | | | | | |
| Quartile 1 | reference | reference | reference |  | reference | reference | reference |  | reference | reference | reference |
| Quartile 2 | 0.80 (0.61 to 1.05) | 0.80 (0.60 to 1.06) | 0.80 (0.61 to 1.07) |  | 0.95 (0.80 to 1.13) | 0.95 (0.80 to 1.13) | 0.95 (0.80 to 1.13) |  | 0.86 (0.72 to 1.04) | 0.86 (0.72 to 1.04) | 0.87 (0.72 to 1.05) |
| Quartile 3 | 0.99 (0.75 to 1.31) | 0.94 (0.71 to 1.24) | 0.94 (0.70 to 1.24) |  | 1.16 (0.98 to 1.38) | 1.16 (0.97 to 1.38) | 1.14 (0.95 to 1.36) |  | 0.95 (0.79 to 1.15) | 0.92 (0.76 to 1.12) | 0.91 (0.74 to 1.11) |
| Quartile 4 | 0.94 (0.71 to 1.24) | 0.93 (0.70 to 1.23) | 0.93 (0.70 to 1.23) |  | 1.01 (0.84 to 1.20) | 1.00 (0.84 to 1.20) | 0.97 (0.81 to 1.17) |  | 1.13 (0.94 to 1.35) | 1.11 (0.93 to 1.34) | 1.10 (0.91 to 1.34) |
| *P* for trend | 0.982 | 0.839 | 0.817 |  | 0.511 | 0.525 | 0.759 |  | 0.112 | 0.168 | 0.248 |
| ***Female (N=3478)*** | | | | | | | | | | | |
| Quartile 1 | reference | reference | reference |  | reference | reference | reference |  | reference | reference | reference |
| Quartile 2 | 0.76 (0.53 to 1.09) | 0.77 (0.53 to 1.11) | 0.78 (0.54 to 1.14) |  | 1.02 (0.81 to 1.30) | 1.02 (0.81 to 1.30) | 1.02 (0.80 to 1.30) |  | 0.95 (0.74 to 1.22) | 0.95 (0.74 to 1.23) | 0.96 (0.73 to 1.25) |
| Quartile 3 | 1.06 (0.75 to 1.48) | 1.02 (0.72 to 1.45) | 0.97 (0.68 to 1.39) |  | 1.05 (0.82 to 1.34) | 1.04 (0.82 to 1.33) | 0.98 (0.76 to 1.25) |  | 1.00 (0.78 to 1.28) | 0.97 (0.75 to 1.26) | 0.89 (0.68 to 1.17) |
| Quartile 4 | 0.92 (0.65 to 1.31) | 0.96 (0.67 to 1.38) | 0.94 (0.65 to 1.35) |  | 1.09 (0.86 to 1.38) | 1.09 (0.86 to 1.39) | 1.01 (0.79 to 1.30) |  | 1.10 (0.86 to 1.41) | 1.14 (0.88 to 1.47) | 1.05 (0.81 to 1.38) |
| *P* for trend | 0.989 | 0.860 | 0.947 |  | 0.476 | 0.457 | 0.985 |  | 0.392 | 0.318 | 0.817 |
| ***Male (N=2983)*** | | | | | | | | | | | |
| Quartile 1 | reference | reference | reference |  | reference | reference | reference |  | reference | reference | reference |
| Quartile 2 | 0.61 (0.38 to 1.01) | 0.61 (0.38 to 1.01) | 0.63 (0.39 to 1.02) |  | 0.91 (0.71 to 1.18) | 0.91 (0.71 to 1.18) | 0.91 (0.70 to 1.17) |  | 0.81 (0.62 to 1.07) | 0.82 (0.62 to 1.07) | 0.84 (0.63 to 1.11) |
| Quartile 3 | 0.96 (0.63 to 1.46) | 0.94 (0.61 to 1.43) | 0.94 (0.61 to 1.44) |  | 1.11 (0.87 to 1.41) | 1.11 (0.87 to 1.42) | 1.11 (0.87 to 1.43) |  | 0.78 (0.59 to 1.02) | 0.77 (0.59 to 1.02) | 0.78 (0.59 to 1.03) |
| Quartile 4 | 0.97 (0.64 to 1.49) | 0.92 (0.60 to 1.41) | 0.93 (0.60 to 1.44) |  | 0.95 (0.73 to 1.22) | 0.95 (0.74 to 1.23) | 0.95 (0.73 to 1.23) |  | 1.16 (0.89 to 1.50) | 1.14 (0.88 to 1.47) | 1.15 (0.88 to 1.50) |
| *P* for trend | 0.719 | 0.972 | 0.968 |  | 0.350 | 0.961 | 0.988 |  | 0.588 | 0.347 | 0.357 |

Notes: CI, confidence interval. MHNW, metabolically healthy with normal weight. MUNW, metabolically unhealthy with normal weight. MHO, metabolically healthy with overweight/obesity. MUO, metabolically unhealthy with overweight/obesity. The reference of multinomial logistic models was MHNW. Model 1 adjusted for sex. Model 2 further adjusted for age based on Model 1. Model 3 further adjusted for smoking history, drinking history, education, residence, marital status, high-sensitivity C-reactive protein, and disease counts based on Model 2.

Supplementary Table 3. Sensitivity analysis: Odds ratios and 95% CIs of different metabolic obesity phenotypes by three iron indicator levels in CHNS 2009.

|  | **MSHNW** | **MUNW** | **MHO** | **MSHO** | **MUO** |
| --- | --- | --- | --- | --- | --- |
|  | **OR (95% CI)** | | | | |
|  | ***Ferritin*** | | | | |
| ***Overall (N=6461)*** | | | |  |  |
| Quartile 1 | reference | reference | reference | reference | reference |
| Quartile 2 | 1.12 (0.91 to 1.37) | 1.35 (0.96 to 1.90) | 1.15 (0.77 to 1.72) | **1.29 (1.03 to 1.61)** | **1.92 (1.49 to 2.48)** |
| Quartile 3 | **1.27 (1.01 to 1.60)** | **1.98 (1.39 to 2.82)** | 1.26 (0.81 to 1.97) | **1.40 (1.09 to 1.80)** | **2.66 (2.03 to 3.50)** |
| Quartile 4 | **1.60 (1.24 to 2.07)** | **4.13 (2.85 to 5.99)** | 1.35 (0.82 to 2.22) | **2.38 (1.81 to 3.14)** | **7.07 (5.28 to 9.47)** |
| *P* for trend | <0.001 | <0.001 | 0.232 | <0.001 | <0.001 |
| ***Female (N=3478)*** | | | |  |  |
| Quartile 1 | reference | reference | reference | reference | reference |
| Quartile 2 | 1.27 (0.98 to 1.66) | 1.01 (0.63 to 1.63) | 1.00 (0.61 to 1.65) | 1.24 (0.93 to 1.65) | **1.43 (1.01 to 2.01)** |
| Quartile 3 | 1.33 (0.98 to 1.79) | 1.53 (0.96 to 2.43) | 1.22 (0.69 to 2.15) | 1.37 (0.99 to 1.90) | **2.01 (1.39 to 2.88)** |
| Quartile 4 | 1.26 (0.89 to 1.80) | **1.67 (1.02 to 2.74)** | 0.73 (0.33 to 1.58) | 1.33 (0.91 to 1.93) | **2.78 (1.87 to 4.13)** |
| *P* for trend | 0.114 | 0.005 | 0.808 | 0.069 | <0.001 |
| ***Male (N=2983)*** | | | |  |  |
| Quartile 1 | reference | reference | reference | reference | reference |
| Quartile 2 | 1.28 (0.97 to 1.70) | 1.67 (0.97 to 2.87) | **1.70 (1.01 to 2.86)** | 1.21 (0.88 to 1.67) | **2.13 (1.47 to 3.09)** |
| Quartile 3 | **1.74 (1.29 to 2.33)** | **2.90 (1.71 to 4.94)** | 1.12 (0.61 to 2.06) | **1.84 (1.32 to 2.56)** | **3.58 (2.46 to 5.20)** |
| Quartile 4 | 1.35 (0.98 to 1.85) | **5.13 (3.07 to 8.59)** | 1.62 (0.90 to 2.90) | **2.11 (1.50 to 2.97)** | **6.83 (4.71 to 9.90)** |
| *P* for trend | 0.006 | <0.001 | 0.256 | <0.001 | <0.001 |
|  | ***Transferrin*** | | | | |
| ***Overall (N=6461)*** | | | |  |  |
| Quartile 1 | reference | reference | reference | reference | reference |
| Quartile 2 | 1.14 (0.93 to 1.38) | **1.49 (1.09 to 2.05)** | 1.06 (0.70 to 1.60) | **1.41 (1.13 to 1.76)** | **1.93 (1.52 to 2.47)** |
| Quartile 3 | **1.38 (1.12 to 1.70)** | **2.30 (1.66 to 3.17)** | **1.78 (1.21 to 2.62)** | **1.94 (1.55 to 2.44)** | **3.14 (2.46 to 4.01)** |
| Quartile 4 | **1.64 (1.32 to 2.03)** | **3.53 (2.55 to 4.87)** | **1.73 (1.14 to 2.61)** | **2.63 (2.08 to 3.32)** | **4.57 (3.56 to 5.87)** |
| *P* for trend | <0.001 | <0.001 | 0.001 | <0.001 | <0.001 |
| ***Female (N=3478)*** | | | |  |  |
| Quartile 1 | reference | reference | reference | reference | reference |
| Quartile 2 | 1.14 (0.86 to 1.52) | **1.66 (1.09 to 2.54)** | 1.19 (0.67 to 2.12) | 1.36 (0.99 to 1.87) | **1.78 (1.27 to 2.50)** |
| Quartile 3 | 1.25 (0.93 to 1.66) | **2.53 (1.66 to 3.86)** | 1.22 (0.68 to 2.18) | **1.69 (1.23 to 2.33)** | **2.64 (1.88 to 3.71)** |
| Quartile 4 | **1.45 (1.08 to 1.95)** | **3.54 (2.30 to 5.45)** | 1.47 (0.82 to 2.61) | **2.43 (1.76 to 3.34)** | **3.65 (2.58 to 5.15)** |
| *P* for trend | 0.013 | <0.001 | 0.206 | <0.001 | <0.001 |
| ***Male (N=2983)*** | | | |  |  |
| Quartile 1 | reference | reference | reference | reference | reference |
| Quartile 2 | **1.40 (1.05 to 1.86)** | 1.59 (0.97 to 2.61) | 1.12 (0.62 to 2.03) | **1.52 (1.09 to 2.11)** | **2.37 (1.64 to 3.44)** |
| Quartile 3 | **1.75 (1.29 to 2.36)** | **2.24 (1.34 to 3.74)** | **2.31 (1.34 to 3.98)** | **2.16 (1.54 to 3.03)** | **4.12 (2.83 to 6.00)** |
| Quartile 4 | **2.14 (1.55 to 2.94)** | **3.82 (2.30 to 6.32)** | **2.21 (1.23 to 4.00)** | **3.17 (2.23 to 4.50)** | **7.73 (5.28 to 11.31)** |
| *P* for trend | <0.001 | <0.001 | <0.001 | <0.001 | <0.001 |
|  | ***Transferrin Receptor*** | | | | |
| ***Overall (N=6461)*** | | | |  |  |
| Quartile 1 | reference | reference | reference | reference | reference |
| Quartile 2 | 0.83 (0.68 to 1.02) | **0.72 (0.53 to 0.98)** | 0.82 (0.56 to 1.21) | 0.86 (0.69 to 1.08) | **0.78 (0.62 to 0.98)** |
| Quartile 3 | 1.06 (0.86 to 1.31) | 0.98 (0.71 to 1.34) | 1.08 (0.73 to 1.62) | 1.20 (0.95 to 1.51) | 0.95 (0.74 to 1.21) |
| Quartile 4 | 1.06 (0.86 to 1.32) | 0.97 (0.70 to 1.33) | 0.96 (0.64 to 1.44) | 1.02 (0.81 to 1.29) | 1.15 (0.91 to 1.46) |
| *P* for trend | 0.196 | 0.662 | 0.796 | 0.260 | 0.083 |
| ***Female (N=3478)*** | | | |  |  |
| Quartile 1 | reference | reference | reference | reference | reference |
| Quartile 2 | 0.84 (0.63 to 1.12) | 0.70 (0.46 to 1.07) | 0.78 (0.45 to 1.35) | 0.95 (0.70 to 1.29) | 0.87 (0.63 to 1.20) |
| Quartile 3 | 1.00 (0.75 to 1.35) | 0.97 (0.65 to 1.47) | 0.95 (0.54 to 1.66) | 0.98 (0.71 to 1.35) | 0.89 (0.64 to 1.24) |
| Quartile 4 | 1.08 (0.80 to 1.44) | 0.99 (0.65 to 1.50) | 0.78 (0.44 to 1.39) | 1.12 (0.81 to 1.53) | 1.12 (0.80 to 1.55) |
| *P* for trend | 0.378 | 0.651 | 0.575 | 0.453 | 0.480 |
| ***Male (N=2983)*** | | | |  |  |
| Quartile 1 | reference | reference | reference | reference | reference |
| Quartile 2 | 1.16 (0.86 to 1.56) | **1.74 (1.03 to 2.93)** | 1.20 (0.69 to 2.10) | 1.21 (0.86 to 1.68) | 1.31 (0.93 to 1.84) |
| Quartile 3 | 1.04 (0.78 to 1.41) | 1.53 (0.90 to 2.58) | 1.03 (0.59 to 1.82) | 1.31 (0.95 to 1.82) | 0.96 (0.68 to 1.36) |
| Quartile 4 | 1.35 (0.99 to 1.83) | **1.79 (1.05 to 3.06)** | 1.37 (0.77 to 2.43) | 1.24 (0.87 to 1.75) | **1.66 (1.17 to 2.35)** |
| *P* for trend | 0.364 | 0.642 | 0.684 | 0.542 | 0.235 |

Notes: OR, odd ratio. CI, confidence interval. MHNW, metabolically healthy with normal weight. MSHNW, metabolically sub-healthy with normal weight. MUNW, metabolically unhealthy with normal weight. MHO, metabolically healthy with overweight/obesity. MSHO, metabolically sub-healthy with overweight/obesity. MUO, metabolically unhealthy with overweight/obesity. The reference of multinomial logistic models was MHNW. OR adjusted for sex, age, smoking history, drinking history, education, residence, marital status, high-sensitivity C-reactive protein, and disease counts.


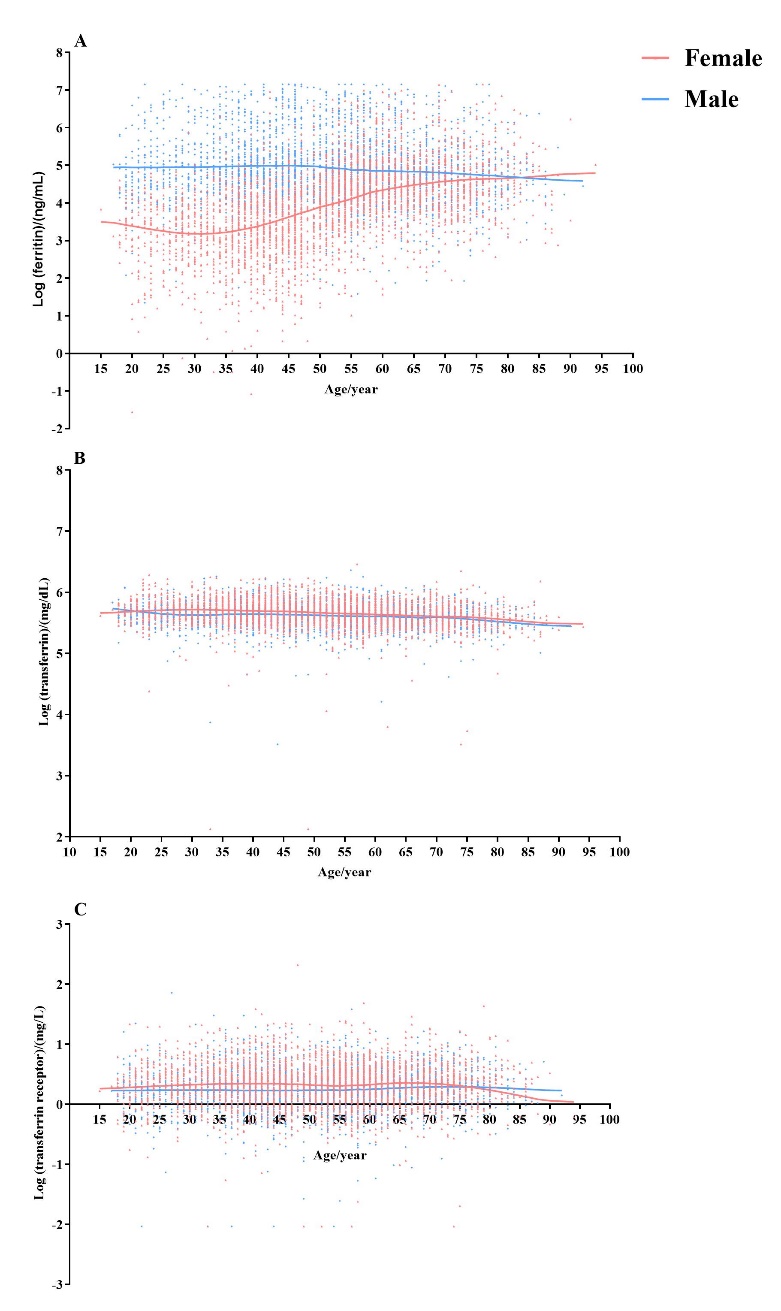


Supplementary Figure 1. The relationships between iron markers and age
